# Effects of atorvastatin on serum lipids, serum inflammation and plaque morphology in patients with stable atherosclerotic plaques

**DOI:** 10.3892/etm.2012.722

**Published:** 2012-09-25

**Authors:** SUXIA GUO, RUXING WANG, ZHENYU YANG, KULIN LI, QIANG WANG

**Affiliations:** Department of Cardiology, Affiliated People’s Hospital of Nanjing Medical University, Chong’an, Wuxi, Jiangsu 214023, P.R. China

**Keywords:** dose-ranging, atorvastatin, serum lipids, serum inflammation, plaque morphology, stable atherosclerotic plaques

## Abstract

Statin treatment in patients with coronary heart disease is associated with a reduced incidence of short-term adverse events and endpoint cardiac events. However, the effects of statin treatment on atherosclerotic plaques, particularly stable plaques, remain poorly defined. In total, 228 consecutive patients with stable atherosclerotic plaques who had undergone coronary arteriography (CAG) and intravascular ultrasound (IVUS) were randomly assigned to receive placebo (placebo group, n=54) or atorvastatin (ATOR) at a single daily dose of 10 mg (ATOR 10 mg group, n=47), 20 mg (ATOR 20 mg group, n=45), 40 mg (ATOR 40 mg group, n=43) or 80 mg (ATOR 80 mg group, n=39). Endpoints, including serum lipids, serum inflammation, plaque volume and percentage of plaque necrosis were assessed after 3–6 months. At baseline, mean low-density lipoprotein (LDL), high-density lipoprotein (HDL) and high-sensitivity C-reactive protein (hs-CRP) levels, as well as plaque volumes and percentages of plaque necrosis, were similar between all groups. At 6 months of follow-up, the LDL levels in the ATOR groups were below those at their respective baselines (P<0.01). HDL levels in the ATOR 80 mg group following treatment were significantly higher compared with baseline (P=0.001). Additionally, they were significantly higher compared with those in the placebo, ATOR 10, 20 and 40 mg groups (P<0.01, P=0.001, P=0.048, P=0.047, respectively). Hs-CRP levels in the placebo group following treatment were higher compared with baseline levels (6.87±2.62 vs. 5.07±1.80, P<0.01), but hs-CRP levels in the ATOR 80 mg group following treatment were lower compared with baseline (3.59±1.07 vs. 6.10±2.12, P<0.01). According to the virtual histology (VH) of IVUS, the percentages of plaque necrosis following treatment in the placebo and ATOR 10 mg groups rose above baseline levels (15.51±12.56 vs. 7.69±1.31%, 13.54±11.76 vs. 7.83±1.43%, P<0.01) and conformed to the diagnostic criteria for unstable plaques (15.51±12.56, 13.54±11.76%). By contrast, in the ATOR 20, 40 and 80 mg groups, percentages of plaque necrosis remained stable following treatment compared with baseline (P=0.069, 0.846 and 0.643, respectively). Plaque volumes following treatment in the placebo, ATOR 10 and 20 mg groups were similar to baseline levels. However, in the ATOR 40 and 80 mg groups, plaque volumes decreased following treatment compared with baseline plaque volumes (30.69±8.12 vs. 37.09±12.01 mm^3^, 24.99±1.01 vs. 36.47±14.68 mm^3^, P=0.019, P<0.01, respectively). ATOR (20 mg/day) is able to lower LDL to standard levels while ATOR 40 mg/day was superior to 20 mg/day and had similar effects to 80 mg/day. Only ATOR 80 mg/day was able to increase HDL levels. Hs-CRP in patients without ATOR was higher and ATOR 80 mg/day decreased levels. ATOR ≥20 mg/day is able to stabilize plaques and ATOR 80 mg/day was superior to 20 and 40 mg/day. Thus, ATOR 40–80 mg/day reduces the volume of plaques.

## Introduction

Increasing evidence has shown that early and aggressive statin therapy decreases the risk of acute myocardial infarction (MI) and major adverse cardiovascular events (MACE) in patients with coronary heart disease (1–3). Several previous studies have also shown that cardiovascular morbidity and mortality in patients with hypercholesterolemia are significantly reduced by statins (4,5). A meta-analysis of six trials in patients with stable angina revealed that statin pretreatment resulted in a 59.3% reduction of relative risk of procedural MI and a 20.5% overall reduction in MACE (6). Several studies have shown various loading doses of atorvastatin (ATOR) therapy prior to percutaneous coronary intervention to be associated with a reduced risk of MACE (7,8). However, the effects of various loading doses of statins in patients with stable atherosclerotic plaques have not yet been evaluated. For this reason, a clinical follow-up study of various loading doses of ATOR on serum lipids, inflammation and plaque morphology in patients with stable atherosclerotic plaques was conducted.

## Patients and methods

### Study population and design

**Figure 1.**
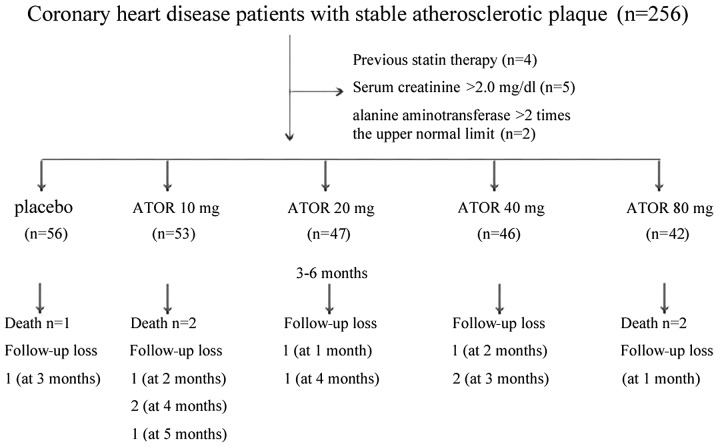
Study design. ATOR, atorvastatin.

The patients included in this study were recruited from Wuxi People’s Hospital, Wuxi City, China, between May 2008 and December 2010. This study was conducted in accordance with the declaration of Helsinki and with approval from the Ethics Committee of Wuxi People’s Hospital, Wuxi City, China. Written informed consent was obtained from all participants. In total, 256 consecutive patients with stable atherosclerotic plaques who had undergone diagnostic coronary angiography and intravascular ultrasound (IVUS) were screened. A total of 11 patients were excluded due to previous statin therapy, renal insufficiency (serum creatinine >2.0 mg/dayl) or hepatic disease (history of liver cirrhosis or alanine aminotransferase greater than twice the upper limit of normal). Eligible patients were randomly assigned to receive no statin treatment (placebo group) or to receive ATOR at a dosage of 10 mg (ATOR 10 mg), 20 mg (ATOR 20 mg), 40 mg (ATOR 40 mg) or 80 mg (ATOR 80 mg). Following six months, five patients had succumbed to the disease and nine patients were lost to follow-up. These patients were excluded and the remaining 228 patients were enrolled in the current study. The study took place after consent was obtained from all patients. The study design is shown in Fig. 1.

Aspirin (100 mg/day) was prescribed to all patients in this study. CK-MB and troponin T levels were measured prior to coronary angiography and IVUS. Additional cardiac enzyme measurements were obtained if the patients revealed signs or symptoms of myocardial ischemia. Low-density lipoprotein (LDL)-cholesterol, high-density lipoprotein (HDL)-cholesterol and high-sensitivity C-reactive protein (hs-CRP) levels were assessed prior to coronary angiography and IVUS. In all patients, angiotensin-converting enzyme inhibitor (ACEI), angiotensin receptor blocker (ARB) and beta blockers were administered according to blood pressure and heart rate.

Patients were followed up for 3–6 months at one-month intervals, through out-patient contact or by telephone. Ten patients were lost to follow-up prior to the end of the full 6-month follow-up period, but their data until this point were included in the statistical analysis. All patients provided written informed consent.

### Coronary angiography and IVUS analysis

**Figure 2:**
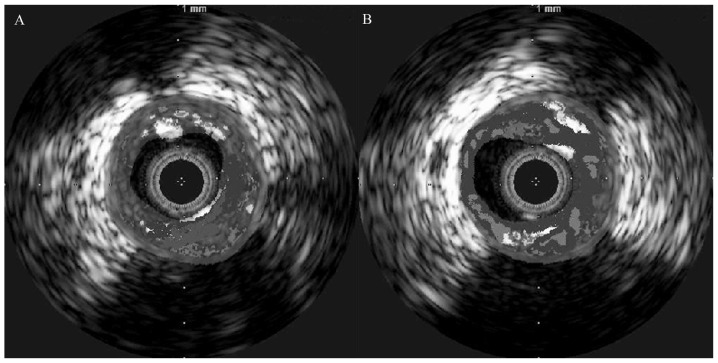
Intravascular ultrasound (IVUS) of two different plaques. (A) Unstable plaque. (B) Stable plaque.

Coronary angiography and IVUS were performed during inpatient treatment (9). Within 3–6 months of coronary angiography, the site was selected for IVUS analysis of the coronary artery (10–12). All IVUS images were acquired using a 20-MHz Volcano Eagle Eye™ IVUS catheter (Volcano Therapeutics Inc,. Rancho Cordova, CA, USA). Once the coronary lesion had been identified, the IVUS catheter was inserted distal to the lesion and manually pulled back to assess the severity and length of the lesion. The IVUS catheter was then placed distal to a side branch (distal fiduciary landmark site) and automatic pullback was performed at a rate of 0.5 mm/sec. The location of the IVUS catheter was determined using continuous fluoroscopy throughout the time of pullback and by recording anatomical landmarks observed during IVUS imaging. To create adequate images, an average of 2 pullbacks per artery were performed and the best play loop was selected based on imaging resolution and quality. Continuous EKG monitoring was performed during the procedure to gate IVUS frames for analysis. IVUS-virtual histology (VH) data were recorded to the imaging system hard drive and then extracted and archived for analysis. Analysis was based on border contour calculation from grayscale. The tissue maps provided by the software (dark green for fibrous tissue, light green for fibrofatty tissue, red for necrotic core and white for dense calcium) were used to analyze each independent frame. Once the total length of each lesion had been determined, a 20-mm vascular segment containing the vascular lesion was selected for analysis. This segment was then divided into equal 2.0-mm subsections, generating a total of 10 series of cross-sections per vascular segment. Unstable and stable plaques are shown in Fig. 2.

### Endpoints

LDL levels <2.06 mmol/l in patients with coronary heart disease were defined as normal. HDL levels >1.0 mmol/l and hs-CRP levels <8 mg/l were defined as normal. Primary endpoints included changes in LDL, HDL and hs-CRP levels from baseline following 3–6 months of no treatment or ATOR treatment at the specified doses. Secondary endpoints included changes in the percentages of plaque necrosis and in plaque volumes. Stable plaques were defined as plaques with <10% necrotic tissue. Plaque volumes = ∑[(external elastic membrane cross-sectional area - lumen cross-sectional area)/the number of sections] x plaque length (3).

### Statistical analyses

All measurements are shown as mean ± standard deviation. Continuous variables between two groups were compared by independent t-tests and Chi-squared tests and multiple groups were compared by ANOVA. All tests were conducted using SPSS 17.0 software for Windows (Lei An Technology Company, Beijing City, China). Proportions were compared using Fisher’s exact test when the expected frequency was <5 and Chi-squared testing in all other cases. P<0.05 was considered to indicate a statistically significant result.

## Results

### Baseline characteristics

There were no significant differences between the five study groups in baseline and other clinical characteristics as shown in Table I. ATOR loading was performed for 3–6 months. VH of IVUS was used with procedural success achieved in all patients. Medication use was similar among groups, including the use of ACEI/ARB and beta blockers.

### Primary endpoints

**Figure 3:**
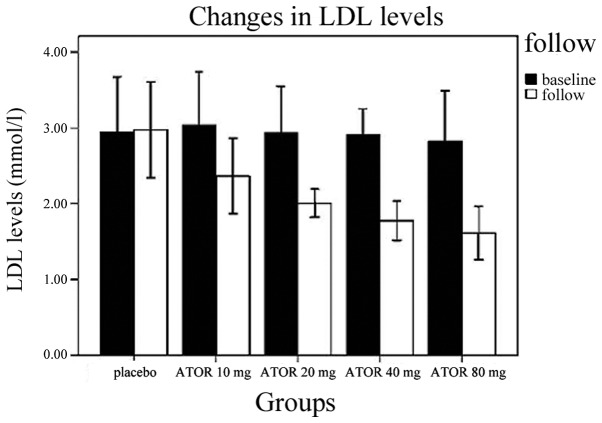
Changes in LDL at baseline and follow-up. LDL, low-density lipoprotein; ATOR, atorvastatin.

During the follow-up period (3–6 months, mean 4.51±1.23), the endpoints of LDL, HDL and hs-CRP levels in the five treatment groups revealed significant differences from baseline. Changes in serum lipids and serum inflammation in groups are shown in Table II. LDL levels at follow-up in the placebo group demonstrated no change over baseline (P=0.813), but LDL levels at follow up in the ATOR 10, 20, 40 and 80 mg groups were lower than their respective baseline levels (all P<0.01). LDL levels in the ATOR 20 mg group at follow-up were statistically significantly higher than in the ATOR 40 mg (P=0.048) and ATOR 80 mg groups at follow-up (P=0.001) and LDL levels in the ATOR 40 mg group at follow-up were similar to the ATOR 80 mg group (P=0.168). Changes in LDL at baseline and follow-up are shown in Fig. 3. HDL levels were significantly higher in the ATOR 80 mg group following treatment than at baseline (P=0.001). HDL levels were also significantly higher in the ATOR 80 mg group following treatment than in the placebo, ATOR 10, 20 or 40 mg groups (P<0.01, P=0.001, P=0.048 and P=0.047, respectively).

**Figure 4:**
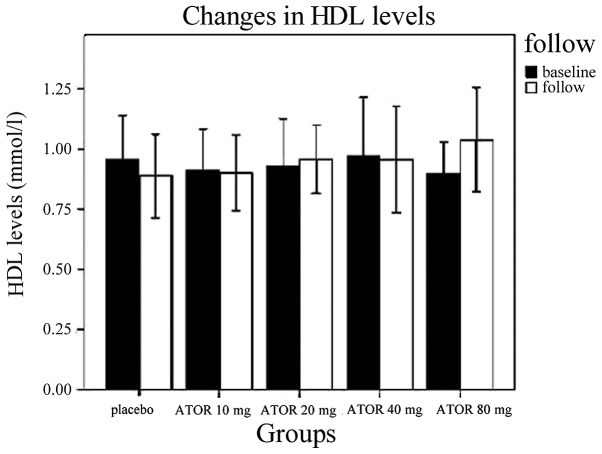
Changes in HDL at baseline and follow-up. HDL, high-density lipoprotein; ATOR, atorvastatin.

**Figure 5:**
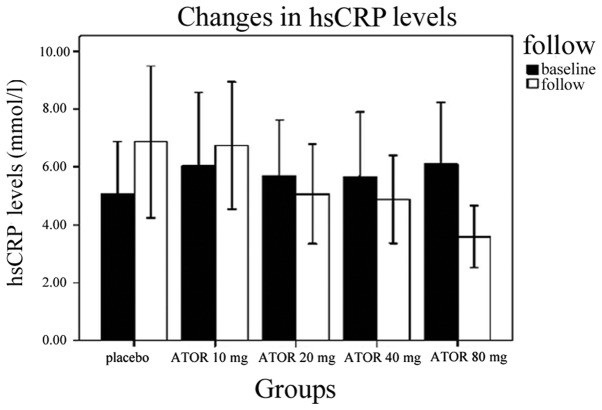
Changes in hs-CRP at baseline and follow-up. Hs-CRP, high-sensitivity C-reactive protein; ATOR, atorvastatin.

However, there were no significant differences between HDL levels amongst the placebo, ATOR 10, 20 or 40 mg groups. Changes in HDL at baseline and follow-up are shown in Fig. 4. Hs-CRP levels at follow-up in the placebo group were higher than baseline (6.87±2.62 vs. 5.07±1.80, P<0.01), while those in the ATOR 80 mg group following treatment were lower than at baseline (3.59±1.07 vs. 6.10±2.12, P<0.01). There were no statistically significant differences between hs-CRP following treatment than at baseline for the ATOR 10 mg, ATOR 20 mg and ATOR 40 mg groups, but higher dosages of ATOR were generally associated with trends toward maintaining or decreasing hs-CRP levels over time with treatment. Changes in hs-CRP at baseline and follow-up are shown in Fig. 5.

### Secondary endpoints

**Figure 6:**
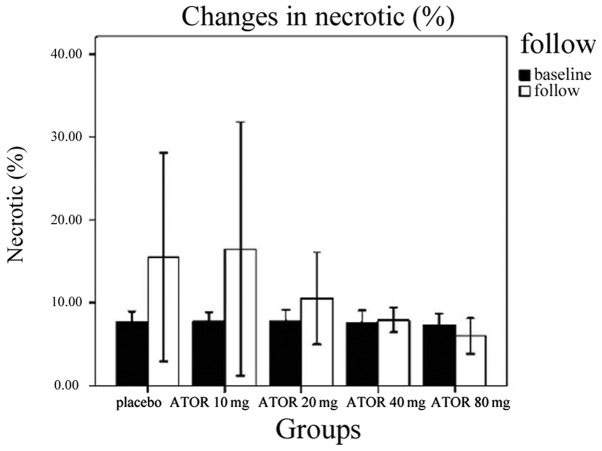
Changes in the relative amount of necrotic tissue at baseline and follow-up. ATOR, atorvastatin.

**Figure 7:**
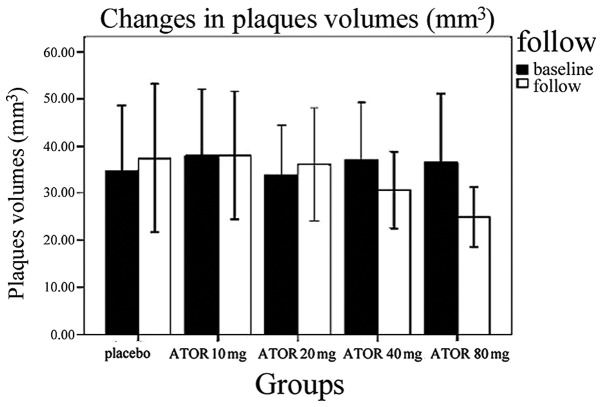
Changes in plaque volume at baseline and follow-up. ATOR, atorvastatin.

According to the VH of IVUS, the percentages of plaque necrosis on follow-up increased in the placebo and ATOR 10 mg groups, compared with baseline percentages (15.51±12.56 vs. 7.69±1.31%, 13.54±11.76 vs. 7.83±1.43%, P<0.01), satisfying the diagnostic criteria for unstable plaques. In the ATOR 20, 40 and 80 mg groups no differences in percentages of plaque necrosis from baseline were observed (P=0.069, 0.846, 0.643, respectively). In the placebo, ATOR 10 and 20 mg groups, plaque volumes did not increase relative to their respective baselines. By contrast, in the ATOR 40 and 80 mg groups, plaque volumes decreased relative to their respective baselines (30.69±8.12 vs. 37.09±12.01 mm^3^, 24.99±1.01 vs. 36.47±14.68 mm^3^, P=0.019, P<0.01). Changes in the percentages of plaque necrosis and plaque volumes in groups are shown in Table III, Figs. 6 and 7

## Discussion

In the present dose-ranging study of ATOR in patients with stable coronary atherosclerotic plaques, the dose-dependent effects of ATOR on serum lipids, serum inflammation and plaque morphology were demonstrated. In general, higher dosages of ATOR up to 80 mg per day for up to 3–6 months of treatment were associated with a greater potential for beneficial effects by decreasing LDL, increasing HDL, decreasing hs-CRP, preventing plaque necrosis and reducing plaque volume in patients with stable coronary atherosclerotic plaques.

Previous studies have demonstrated that statin therapy improves the prognosis of patients with coronary heart disease (13–15). The PROVEIT study reported that a dose of 80 mg ATOR per day reduced the risk of negative outcomes at 30 days and 2 years (16). The MIRACL study revealed that 80 mg ATOR per day reduced the risk of the composite primary endpoints comprising mortality, MI, cardiac arrest and recurrent ischemia (17). The study indicated that high, early doses of statin therapy significantly improved the prognosis in patients with heart disease. A meta-analysis of six trials (6) in patients with stable angina revealed that statin pretreatment was associated with a 59.3% reduction in the relative risk of procedural MI and a 20.5% overall reduction in MACE. Several studies have been performed to evaluate the benefits of high doses of statins in patients with heart disease (18–20). However, to date, no studies have evaluated the effects of various doses of ATOR on stable atheroscletotic plaques.

In the current study, the age, male-to-female ratio, history of diabetes and hypertension, history of alcohol use and smoking, pre-treatment medication regimen (including ACEI, ARB and beta blockers), as well as pre-treatment serum lipid levels and hs-CRP levels, of the patients were not significantly different between the five groups.

Following treatment, the LDL, HDL and hs-CRP levels differed among the five groups. For LDL, the use of ATOR at 20 mg/day brought LDL levels down to the standard value, while 40 and 80 mg/day were identified to be similar to each other and more effective than 20 mg/day in lowering LDL levels. Treatment with 80 mg/day increased HDL levels and no effect was observed with any of the lower dosages. In patients who were not administered statins, the hs-CRP levels increased relative to baseline, but 80 mg/day ATOR lowered hs-CRP levels. Doses of 20 mg/day and higher kept plaques stable as demonstrated by prevention of progressive plaque necrosis with treatment, compared with no treatment. In addition, 80 mg/day was revealed to be significantly more effective than 20 or 40 mg/day at decreasing plaque volume. This indicated dose-dependent effects of ATOR on clinically relevant serum lipid and inflammatory markers as well as in plaque morphology in patients with stable atherosclerotic plaques.

The current study has several limitations. Firstly, the study was not blinded. Secondly, the sample size was relatively small. Finally, the duration of follow-up was relatively short. Of note, our original study design called for two years of follow-up, but the study was stopped early due to statistically significant results being obtained on interim analysis after 3 and 6 months of follow-up. Further study is required to confirm our results, ideally in an independent sample population.

In conclusion, ATOR at a dosage of 80 mg/day for 6 months is associated with improvements in the serum lipid profile, decrease in serum inflammation and the maintenance of plaque stability in patients with stable coronary atherosclerotic plaques. Several of these potentially beneficial effects were also observed at lower dosages of ATOR, but these effects were more marked and consistent at 80 mg/day.

## Figures and Tables

**Table I:** Baseline clinical characteristics.

Characteristics	Placebo (n=54)	ATOR 10 mg group (n=47)	ATOR 20 mg group (n=45)	ATOR 40 mg group (n=43)	ATOR 80 mg group (n=39)	F(χ^2^) P-value
Age (years)	62.07±8.51	62.64±12.00	59.18±8.48	58.91±12.90	58.95±9.68	9.085 (0.059)
Male/female	48/6	40/7	36/9	41/2	34/5	5.00 (0.288)
FBG (mmol/l)	5.26±0.98	5.73±1.00	5.72±0.82	5.00±0.83	5.63±0.97	0.010 (0.922)
Alcohol (yes/no)	33/21	31/16	25/20	26/17	20/19	2.246 (0.690)
Smoker (yes/no)	46/8	35/8	35/10	37/6	32/7	2.962 (0.564)
LVEF (%)	53.65±11.69	55.15±13.16	58.09±11.10	57.14±10.34	55.67±10.96	1.095 (0.360)
Creatinine (mg/dl)	85.11±21.63	87.33±15.07	81.57±16.93	83.86±12.90	88.11±15.28	1.061 (0.377)
ACEI (yes/no)	38/16	31/16	31/14	33/10	23/16	3.027 (0.524)
Beta-blocker (yes/no)	28/26	29/18	21/24	21/22	15/24	4.927 (0.295)

FBG, fasting blood glucose; LVEF, left ventricular ejection fraction. ATOR, atorvastatin; ACEI, angiotensin-converting enzyme inhibitor.

**Table II:** Changes in serum lipids and serum inflammation in the study groups.

Variable	Placebo (n=54)	ATOR 10 mg group (n=47)	ATOR 20 mg group (n=45)	ATOR 40 mg group (n=43)	ATOR 80 mg group (n=39)
HDL (mmol/l)					
Baseline	0.96±0.18	0.90±0.17	0.93±0.20	0.97±0.24	0.90±0.13
Follow-up	0.89±0.17	0.90±0.16	0.96±0.14	0.96±0.22	1.03±0.22
LDL (mmol/l)					
Baseline	2.94±0.72	3.03±0.70	2.92±0.62	2.90±0.34	2.83±0.66
Follow-up	2.97±0.63	2.36±0.50	2.01±0.18	1.85±0.22	1.81±0.32
Hs-CRP (mg/l)					
Baseline	5.07±1.80	6.04±2.52	5.09±1.94	5.67±2.22	6.10±2.12
Follow-up	6.87±2.62	6.74±2.20	5.07±1.72	4.88±1.52	3.59±1.07

Values are expressed as mean ± standard deviation. HDL, high-density lipoprotein; LDL, low-density lipoprotein; hs-CRP, high-sensitivity C-reactive protein; ATOR, atorvastatin.

**Table III:** Changes of the percentages of plaque necrosis and plaque volumes in groups.

Variable	Placebo (n=54)	ATOR 10 mg group (n=47)	ATOR 20 mg group (n=45)	ATOR 40 mg group (n=43)	ATOR 80 mg group (n=39)
Necrotic (%)					
Baseline	7.69±1.31	7.83±1.03	7.91±1.27	7.64±1.44	7.38±1.33
Follow-up	15.51±12.65	16.54±15.76	10.55±5.56	7.93±1.49	6.66±1.92
Plaque volume (mm^3^)					
Baseline	34.83±13.76	38.07±13.94	33.83±10.56	37.06±12.01	36.47±14.68
Follow-up	37.46±15.80	38.05±13.56	36.12±11.96	30.69±8.12	24.99±1.01

Values are expressed as mean ± standard deviation. ATOR, atorvastatin.
